# Phenotypic and functional analysis of monocyte populations in cattle peripheral blood identifies a subset with high endocytic and allogeneic T-cell stimulatory capacity

**DOI:** 10.1186/s13567-015-0246-4

**Published:** 2015-09-25

**Authors:** Yolanda Corripio-Miyar, Jayne Hope, Colin J McInnes, Sean R Wattegedera, Kirsty Jensen, Yvonne Pang, Gary Entrican, Elizabeth J Glass

**Affiliations:** Division of Infection & Immunity, The Roslin Institute and Royal (Dick) School of Veterinary Studies, University of Edinburgh, Easter Bush, Midlothian, EH25 9RG UK; Current address: Moredun Research Institute, Pentlands Science Park, Bush Loan, Midlothian, EH26 0PZ UK

## Abstract

Circulating monocytes in several mammalian species can be subdivided into functionally distinct subpopulations based on differential expression of surface molecules. We confirm that bovine monocytes express CD172a and MHC class II with two distinct populations of CD14^+^CD16^low/-^CD163^+^ and CD14^−^CD16^++^CD163^low-^ cells, and a more diffuse population of CD14^+^CD16^+^CD163^+^ cells. In contrast, ovine monocytes consisted of only a major CD14^+^CD16^+^ subset and a very low percentage of CD14^−^CD16^++^cells. The bovine subsets expressed similar levels of CD80, CD40 and CD11c molecules and mRNA encoding CD115. However, further mRNA analyses revealed that the CD14^−^CD16^++^ monocytes were CX3CR1^high^CCR2^low^ whereas the major CD14^+^ subset was CX3CR1^low^CCR2^high^. The former were positive for CD1b and had lower levels of CD11b and CD86 than the CD14^+^ monocytes. The more diffuse CD14^+^CD16^+^ population generally expressed intermediate levels of these molecules. All three populations responded to stimulation with phenol-extracted lipopolysaccharide (LPS) by producing interleukin (IL)-1β, with the CD16^++^ subset expressing higher levels of IL-12 and lower levels of IL-10. The CD14^−^CD16^++^ cells were more endocytic and induced greater allogeneic T cell responses compared to the other monocyte populations. Taken together the data show both similarities and differences between the classical, intermediate and non-classical definitions of monocytes as described for other mammalian species, with additional potential subpopulations. Further functional analyses of these monocyte populations may help explain inter-animal and inter-species variations to infection, inflammation and vaccination in ruminant livestock.

## Introduction

The innate immune system is the first line of host defense against pathogens, playing an important role during the early phase of infection. Myeloid cells are among the key mediators of the innate immune system and consist of heterogeneous populations with overlapping relationships and function between monocytes, macrophages and dendritic cells (DC) [1-3]. These populations differ phenotypically and functionally from each other based on their tissue location and previous environmental history [1-5]. Myeloid cells link the innate immune response to the ensuing adaptive immune response as antigen presenting cells. However, what is less clear is the relative contribution of different subsets of myeloid cells, namely monocytes, macrophages and DC in vivo to T cell priming, modulating and directing the quality of the elicited immune response or their precise role in inducing pathology or protection [2,6]. It is likely that different myeloid subsets are important for controlling different pathogens. Consequently, one way to improve the efficacy of vaccines is to identify and target the myeloid subsets that are important for driving immune responses in appropriate directions.

Historically, most research into myeloid cells has concentrated on cell subsets derived from mouse tissue and, to a lesser extent, human peripheral blood monocytes, including cells that have been differentiated in vitro. It is not entirely clear how these subsets in different species relate to each other, or how relevant in vitro derived myeloid cells are to the cells observed in specific tissue locations in vivo. However in general, the consensus is that in humans and mice, monocytes can be divided into two distinct subpopulations together with a third less well defined intermediate subpopulation [4]. These subpopulations appear to be phenotypically and functionally similar between the two species and are distinguished on the basis of CD14 and CD16 (FcγRII) expression in humans and Ly6C and CD43 in mice [7,8]. The major human monocyte population is referred to as “classical” and is CD14^++^CD16^−^ (Ly6C^++^CD43^+^ in the mouse) and the minor is a more mature human population referred to as “non-classical” which is CD14^+^CD16^++^ (Ly6C^+^CD43^++^ in the mouse). The latter represents around 10% of the total monocyte population [9]. The intermediate population likely represents gradual development from classical to non-classical monocytes, thus delineating this population by the expression levels for these markers can be difficult and it has been recommended that threshold expression levels should be adopted with reference to isotype controls [4]. However some authors consider that the intermediate monocytes and the non-classical CD14^+^CD16^++^ monocytes form a single population, even though phenotypic and gene expression differences between these populations have been reported [2]. Although these different monocyte populations show distinct phenotype and function [2–4], there is still controversy over the precise role of each of these subsets in inflammatory conditions [4,10]. The evidence derived mainly from mouse studies suggests that the classical monocyte population responds to cytokine and chemokine signals by entering sites of infection and differentiating into macrophages and dendritic cells, thus contributing to inflammation and resolution of the infection [2]. These activities are reflected in human classical monocyte responses to TLR ligands which result in pro-inflammatory cytokine up-regulation, accompanied by release of interleukin (IL)-10, although some studies suggest the intermediate monocyte population is the major IL-10 producing subset [11]. In contrast the non-classical population appears to be mainly involved in patrolling the endothelium of the blood vessels, expressing very little IL-10 and with high levels of the pro-inflammatory cytokine tumour necrosis factor-alpha (TNF-α) [12,13].

It is becoming clearer that rodents are not always the most suitable models for human immunological studies as their immune repertoire and physiology is distinct [14–16]. Indeed in comparing artiodactyla, primates and rodents in terms of evolution of codon usage, rodents are revealed to be an outgroup [17] and their immune responses would also suggest closer relationships exist between these larger mammals compared to rodents [14,18–20]. Nonetheless, it is also important to note that livestock species are affected by overlapping, zoonotic and distinct pathogens resulting in differential evolutionary selective pressure on host immune genes and pathways [21–23]. Indeed, although porcine monocyte subsets have similar characteristics to human classical and intermediate populations, there does not appear to be a non-classical equivalent [18]. Thus a broader exploration of the range of species differences in immune cells and mechanisms has the potential to shed light on our understanding of the diversity of innate immunity and evolution of host-pathogen interactions as livestock species offer the opportunity to study host responses to pathogens within the natural host [19,24–26]. Together with the capability to access different tissue compartments, these larger mammalian species provide alternative models to explore monocyte/macrophage relationships in health and disease.

Current knowledge of myeloid cell lineages and functional specialisation in ruminants is limited. CD16 has previously been reported to be expressed on natural killer (NK) cells in cattle [27] and sheep [28], while CD14 is characteristically expressed on monocytes/macrophages in ruminants [29,30]. A recent report by Hussen et al. [31] has suggested that, in contrast to findings in humans and mice, a bovine non-classical CD14^+^CD16^+^ population exists in peripheral blood monocytes and is relatively non-inflammatory.

Here, we focus on extending the phenotypic and functional characterisation of myeloid cell populations in the peripheral blood of cattle as a basis for exploring their relationships to myeloid cells trafficking into sites of infection and their role in pathogen and vaccine responses.

## Materials and methods

### Animals

Healthy Holstein-Friesian cattle were maintained at The Roslin Institute (RI), UK. In some experiments calves of defined MHC class I haplotype [32,33] were used. All cattle were animals under 2 years of age and kept off pasture. Healthy Texel-Greyface sheep were derived from the breeding stock at the Moredun Research Institute (MRI) and kept off pasture. All experiments were approved by Ethics Committees at RI and MRI and were performed to Home Office Guidelines under Project Licences (PPL 60/4394 and PPL 60/3854 respectively).

### Flow cytometric analysis

**Figure 1:**
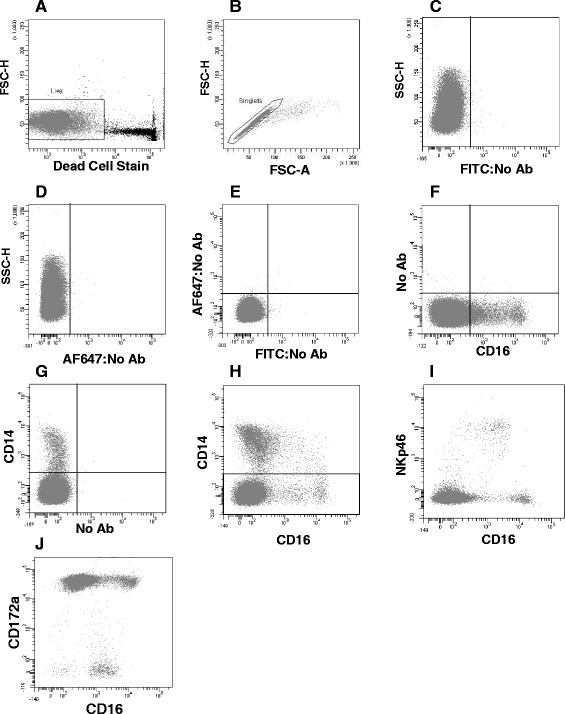
**Gating strategies and myeloid population identification.** The expression of CD16 and CD14 was determined by single and two colour flow cytometry in freshly isolated bovine PBMC. The cells were gated to eliminate dead cells (**A**) and doublets (**B**). Threshold levels which determined positivity for CD14 and CD16 were set with no antibody control (**C**, **D** and **E**) and FMO controls (**F**, **G**) were used to determine quadrant position and fluorescence intensity for subsequent analysis. Cells above fluorescence of 400 for FITC (CD16) and above 300 for AF647 (CD14) were determined as positive for the respective molecules. In order to further define the CD16^+^ populations triple staining with CD14, CD16 and NKp46 (**I**) or CD14, CD16 and CD172a (**J**) was carried out. Within PBMC gated as CD14^−^CD16^+/++^ (rectangular gate; **H**) the majority of NKp46^+^ NK cells expressed CD16 at a fluorescence intensity of ~800-10 000 (**I**). The majority of cells which were CD172a negative (**J**) expressed CD16 at fluorescence intensity ~800-10 000 corresponding with the NKp46^+^ population. A subpopulation of CD14^−^ cells which were NKp46^−^ and CD172a^+^ were observed at fluorescence intensities ≥10 000 and were gated in further studies as a separate population for analysis. Data shown are for one representative animal out of six.

Single or multiple colour flow cytometric analyses were carried out on peripheral blood mononuclear cells (PBMC) from both sheep and cattle. Blood was collected aseptically into blood bags containing 70 mL of citrate phosphate dextrose-adenine 1 (CPDA-1) stabiliser (Sarstedt, Germany) for cattle or in sodium heparin vacutainers (Becton Dickinson, Oxford, UK) for sheep. PBMC were separated by density gradient centrifugation onto Lymphoprep (Axis-Shield, Scotland, UK) for sheep, washed three times with phosphate buffered saline (PBS) and re-suspended at 2 × 10^7^ cells/mL in PBS supplemented with 0.5% foetal bovine serum (FBS, from Brazil supplied by Gibco, Life Technologies, USA) ready for staining. Flow cytometry was carried out using monoclonal antibodies (mAb) to the molecules detailed in Table 1 on 10^6^ cells per antibody combination at pre-optimised concentrations. When primary mAbs were unconjugated, isotype-specific secondary mAb conjugated to phycoerythrin (PE) (Invitrogen, Life Technologies, USA) was used. Finally, cells were resuspended in the dead cell stain Sytox Blue (Invitrogen, Life Technologies, USA) prior to analysis in flow cytometer. Dead cell and doublet cell discrimination (Figure 1A and B) was carried out during analysis of the phenotyping and phagocytosis studies, and Fluorescence Minus One (FMO) controls were used in multiple colour flow cytometry (Figure 1C-E). Cells were initially stained for CD14, CD16, CD172a and NKp46 to ascertain the major myeloid populations in PBMC and to measure CD16 levels on NK cells enabling exclusion from further analyses (Figure 1F-J). Phenotypes were expressed as the geometric mean fluorescence intensity (MFI) and/or % positivity and were collected from six animals.

**Table 1:** **Antibodies list**

**Antigen**	**Antibody nomenclature**	**Isotype**	**Conjugate**	**Source**
CD1b	CC14	IgG1	Nil	IAH, AbD Serotec
CD80	IL-A159	IgG1	Nil	ILRI, AbD Serotec
CD86	IL-A190	IgG1	Nil	ILRI, AbD Serotec
CD40	IL-A156	IgG1	Nil	ILRI, AbD Serotec
MHCII-DR	CC108	IgG1	Nil	IAH, AbD Serotec
CD11b	CC94	IgG1	Nil	IAH
CD11c	NAM4	IgG1	Nil	[44]
CD2	CC42	IgG1	Nil	IAH, AbD Serotec
CD3	MM1A	IgG1	Nil	VMRD, Inc., Pullman, WA
CD4	CC30	IgG2a	R:PE	IAH, AbD Serotec
CD8α	CC63	IgG2a	R:PE	IAH, AbD Serotec
CD8β	CC58	IgG1	R:PE	IAH, AbD Serotec
CD21	CC21	IgG1	Nil	IAH, AbD Serotec
NKp46	GR13.1(EC1.1)	IgG1	Nil	Timothy Connelly (UoE), AbD Serotec
CD26	CC69	IgG1	Nil	IAH
CD172a	CC149	IgG1	Nil	IAH
CD206	3.29B1.10	IgG1	R:PE	Beckman Coulter
TLR2	HuCaL AbD 16476.1		Nil	AbD Serotec
CD163	EDHu-1	IgG1	Nil	AbD Serotec
CD16	KD1	IgG2a	FITC	AbD Serotec
CD14	TUK4	IgG2a	Alexa Fluor 647	AbD Serotec
IgG1 isotype control	AV20	IgG1	Nil	IAH, [35]
IgG2a isotype control	AV29	IgG2a	Nil	IAH, [35]

Monoclonal antibodies used for flow cytometric analysis. IAH, Institute for Animal Health; ILRI, International Livestock Research Institute; UoE, University of Edinburgh.

A minimum of 50 000 events were acquired using an LSRFortessa™ cell analyzer (Becton Dickinson, Oxford, UK) and analysed using FlowJo vX for Windows 7 or FACSDiva v8.0 software.

### Stimulation of purified myeloid cells with lipopolysaccharide (LPS)

A total of 3 × 10^8^ PBMC from six animals were labelled with CD16 and CD14 antibodies and then purified into three populations based on differential expression of CD16 and CD14 (CD14^+^CD16^+^, CD14^+^CD16^low/-^ and CD14^−^CD16^++^) using a FACSAria™ III. After cell sorting, the purities of each population were assessed and only used if the purity was above 85% (Table 2). Cells were washed with PBS and resuspended in RPMI-1640 medium supplemented with 0.1% 2-mercaptoethanol, 1% L-glutamine (mixed leukocyte culture media, MLC) and 10% FBS. Each sorted population was then aliquoted into two wells of a 12-well flat bottom plate (Corning Costar, Sigma-Aldrich, UK) and stimulated for 18 h with 1 μg/mL of phenol-extracted LPS from *E.coli* 055:B5 (L2880; Sigma-Aldrich, UK) or incubated with PBS (control) at 37 °C with 5% CO_2_ in air. After the incubation period, supernatants were obtained by centrifugation and stored at −20 °C until assayed for cytokine production.

**Table 2:** **Details of the oligonucleotides used in the RT-qPCR analysis**

**Gene symbol**	**Accession no.**	**Orientation**	**Sequence (5’-3’)**
CCR2	NM_001194959	F	GATGAAGAACCCACCACCAG
		R	CAAAGATGAAGACCAGCGAGTAG
CD14	NM_174008	F	CGATTTCCGTTGTGTCTGC
		R	TACTGCTTCGGGTTGGTGT
CD16A	NM_001077402	F	TGTCTCGTCATTCTTTCTACCTTG
		R	ACTTTGCCATCCCTCCATTC
CD163	NM_001163413	F	CTTCGGTCCCTTCACTCTTG
		R	CCAGCCTCAGTTCCTTGTCT
CD115	NM_001075403	F	ACCTTGACATTGGAGCCTGA
		R	CGGAAGTCGGATTGTTGAGA
CX3CR1	NM_001102558	F	TCACCAGAGAGAAAGAGAACGA
		R	GGAGCAGGAAGCCAAGAAA
CHAMP1	NM_001205506	F	AGCAGTGACCAAGAGCAGGT
		R	TCATAGCACGACAGCAACAA

F and R denote forward and reverse oligonucleotides respectively.

### ELISA

Capture ELISAs were performed to examine the secretion of selected cytokines. IL-1β and IL-6 ELISAs (Thermo Fisher Scientific, MA, USA) were performed as per the manufacturer’s instructions. Antibodies for IL-10 [34], IL-12 [35] and TNF-α [36] were obtained from AbD Serotec, UK. All ELISAs were performed as follows. All incubations were carried out at room temperature and wash steps were performed 6 times with 350 μl wash buffer (PBS + 0.05% Tween 20) using a Skatron Skanwasher 300. Samples and reagents were applied in 100 μL volumes. High-binding capacity ELISA plates (Maxisorp, Nunc, Denmark) were incubated with coating antibodies overnight at room temperature and then washed and blocked for 1 h. Following a further washing step, cell supernatants and standards were added in duplicate for 1 h. Titrated culture supernatants from COS-7 cells transfected with IL-10, IL-12 or TNF-α were used as standard preparations for the measurement of these cytokines [37]. Subsequently plates were washed, detection antibodies added for 1 h, followed by washing and addition of Streptavidin-HRP for 45 min. After the final washing step, TMB substrate was added and the reaction was stopped by the addition of H_2_SO_4_. Absorbance values were read at 450 nm and 550 nm (background). Since different numbers of cells were obtained in the cell sorts for each population (see Table 2), the OD values were compared to the standard curves and values were then normalised and expressed as the concentration (picograms (pg) or biological units (BU)) relative to 10^6^ cells.

### Mixed leukocyte reaction

In order to determine the T cell stimulatory capability of the cell populations of interest in the context of an allogeneic mixed leukocyte reaction (MLR), cells from two animals with defined homozygous distinct MHC class I haplotypes (A14 and A10) were used [32,33]. PBMC from both animals were isolated as described above and re-suspended to a concentration of 10^6^ cells/mL in MLC with 10% FBS. Stimulator and responder cells were as follows: responder cells were PBMC obtained from an A10 calf. Stimulator allogeneic cells from an A14 (MHC mismatched) calf were purified, irradiated (60Gy) myeloid cells (CD14^+^CD16^+^, CD14^+^CD16^low/-^ and CD14^−^CD16^++^). Responder cells (10^5^ per well) were incubated with 10^4^, 10^3^ or 10^2^ stimulators (ratios of 10:1, 100:1 and 1000:1 respectively) in quadruplicate in U-well microtitre plates in a total volume of 200 μL. Controls consisted of responder or non-irradiated stimulator cells in medium alone or with 5 μg/mL of ConA, and irradiated sorted cells alone. Cells were incubated at 37 °C for 5 days. Proliferation was measured by the incorporation of methyl-3H thymidine (0.5 μCi per well; Amersham Biosciences UK Ltd, Chalfont St. Giles, Buckinghamshire) for the final 18 h of culture [38]. Data are presented as the corrected counts per minute (ccpm) averaged over 3 min.

### Tracer endocytosis

Freshly isolated PBMC from four animals were re-suspended in PBS + 0.5% FBS at a concentration of 10^6^ cells/mL. 100 μL of the cell suspension was incubated with TexasRed-Dextran (Molecular Probes, Life Technologies, USA) or TexasRed-OVA (10 000 MW, Molecular Probes, Life Technologies, USA) at a final concentration of 100 μg/mL for a period of 30 min at 37 °C or on ice in 96-well round-bottom plates. Cells were then washed 3 times with cold PBS and then incubated with fluorochrome-conjugated CD16 and CD14 mAbs. In order to take into consideration the non-specific surface binding of both TexasRed-OVA and TexasRed-Dextran, the MFI from the cells incubated on ice was compared to that of cells incubated at 37 °C. Results for each population are therefore expressed as the corrected MFI at 37 °C of cells gated as CD14^+^ or CD14^−^CD16^++^.

### RT-qPCR analysis

CD14^−^CD16^++^ and CD14^+^ populations were purified from PBMC from four animals as follows. In order to obtain a highly pure CD14^−^CD16^++^ population, PBMC were incubated with FITC conjugated CD16 antibody for 20 min at RT. After two washes, labelled cells were incubated with Anti-FITC MicroBeads (Miltenyi Biotech, Germany) for 30 min at 4 °C following manufacturer’s instructions. Cells were then washed in MACS buffer and loaded onto a LS separation column placed in the MACS magnet. Cells labelled with the complex CD16-FITC:MACS magnetic beads were eluted from the column and washed twice in buffer. Cells enriched for CD16 were then further sorted on the basis of the high level of CD16 expression on monocytes, using a FACSAria™ III, as detailed above, resulting in a pure population of CD14^−^CD16^++^ monocytes. A pure CD14^+^ population was obtained from total PBMC using anti-human CD14 MACS microbeads (Miltenyi Biotech, Germany) as detailed above for the CD16 enrichment. In both cases purity was assessed by flow cytometry to ascertain that purity was higher than 85%. Purified populations were then lysed in TRIzol® (Invitrogen, Life Technologies, US) and total RNA was isolated following the manufacturer’s instructions. Finally, RNA concentrations were quantified using a Nanodrop Spectrophotometer (NanoDrop Technologies, Thermo Fisher Scientific, MA, USA) and quality assessed with an Agilient 2100 Bioanalyzer RNA Kit (Agilient Technologies, Santa Clara, California, USA).

First strand cDNA was reverse transcribed from 100 ng total RNA using oligo(dT) primer and GoScript (Promega, Madison, Wisconsin, USA) according to the manufacturer’s instructions. The qPCR was carried out using the Brilliant III ultra-fast SYBR Green Mastermix kit (Agilent Technologies, Santa Clara, California, USA). Oligonucleotides were designed for each gene using Primer3 [39] and Netprimer (Biosoft International, Palo Alto, California, USA) software (Table 2). Reactions were carried out in 10 μL volumes containing: 6.5ul 1 × SYBR Green Master mix with reference dye, 0.5 μL forward and reverse primers at predetermined optimal concentrations and 2.5 μL cDNA diluted at 1:25 for all genes. Amplification and detection of products was carried out using a Mx3000P PCR machine (Stratagene, Agilient Technologies, USA) with the following cycle profile: 95 °C for 3 min followed by 50 cycles of 95 °C for 10 s and 60 °C for 22 s. The detection of a single product was verified by dissociation curve analysis. Each PCR experiment was carried out in triplicate and contained several non-template controls and a log_10_ dilution series of the representative standard. The relative quantities of mRNA were calculated using the method described by Pfaffl [40]. The results for each target gene were normalized against the results for chromosome alignment maintaining phosphoprotein 1 (CHAMP1), which exhibited the least variation across the samples in a comparison of four house-keeping genes (data not shown). CHAMP1 was previously identified as a constitutively and moderately expressed gene in resting and activated monocytes [41].

### Statistical analyses

Statistical analysis of ELISA data was performed using a General Linear Model (GLM). The ratio of cytokine level in the presence of LPS divided by cytokine level in medium alone (fold increase) data were analysed using a general linear model fitting cell type, animal and control values as fixed effects. The control values were included as covariates in order to increase the sensitivity of the model. Post-hoc pairwise comparisons between cell types were then made using Fisher tests. A similar GLM analysis was conducted to compare cytokine levels in the presence of medium alone across the three myeloid populations, fitting cell type and animal in the model. Differences in the expression of cell surface markers were measured using One-Way ANOVA, whilst the variation in the mRNA levels of monocyte subset markers measured by RT-qPCR was examined by paired *t*-test analysis. All analyses were carried out within the Minitab version 17 statistical package, with *p* < 0.05 considered significant.

## Results

### Ruminant blood contains cell populations with differential expression of CD14 and CD16

**Figure 2:**
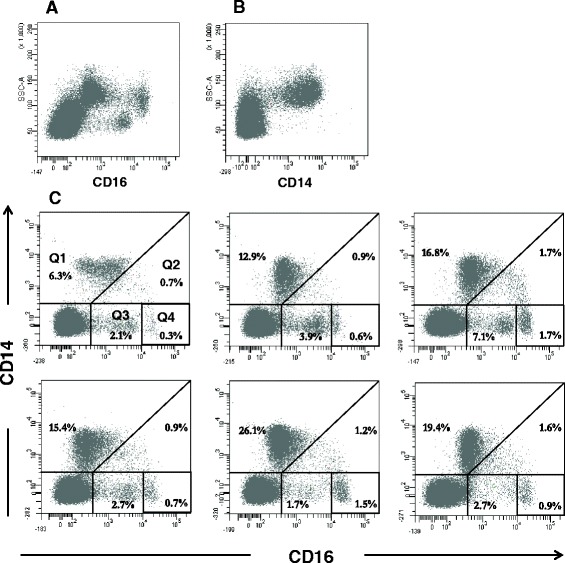
**Expression of CD14 and CD16 defines subpopulations of cells in bovine peripheral blood with differential expression.** The expression of CD16 and CD14 was determined by flow cytometry in freshly isolated bovine PBMC. Live, single cells gated as in Figure 1, were analysed by staining for CD16 (**A**) or CD14 (**B**). To further characterise these populations, live single cells were then assessed for expression of CD16 and CD14 by double staining (**C**). Variable CD16 expression was observed. As shown in Figure 1 cells with CD16 fluorescence <400 were negative for expression. Cells with a CD16 fluorescence between 800 and 10 000 were NKp46^+^ NK cells (panel Q3). Cells with CD16 fluorescence above 10 000 (panel Q4) were identified as a discrete population of CD172a^+^ NKp46^−^ CD16^+^CD14^−^ cells (CD14^−^CD16^++^). CD14^+^ cells had fluorescence intensity >300 and expressed differential levels of CD16. Two CD14^+^ populations were defined by a diagonal gate (panels Q1 and Q2 [8]). Data shown are for one representative animal (**A**, **B**) and for six individual animals (**C**).

In order to identify myeloid cell populations in the peripheral blood of cattle, expression of CD14 and CD16 was analysed on PBMC (*n* = 6). After dead cell and doublet discrimination (Figure 1A and B), a number of CD16 positive sub-populations with different fluorescence intensities and complexity were evident (Figures 1F, 2A); whereas the expression of CD14 was more uniform with a major population observed (Figures 1G, 2B). In order to further define the nature of the CD14^+^ and CD16^+^ populations, PBMC were double-labelled with anti-CD14 and anti-CD16 conjugated antibodies, a method commonly used to identify monocyte subsets in human blood [2,7,9,42]. Although considerable variation in the staining patterns was observed across the six cattle studied (Figure 2C), there was evidence for the presence of a number of sub-populations of cells with differential CD14 and CD16 expression. Within the CD14 negative population there were cells with varying levels of CD16 expression. We demonstrated that CD14^−^ cells expressing CD16 at MFI of ~800-10 000 (Figure 2C, panel Q3) were NK cells as these expressed NKp46 (Figure 1I) and were negative for CD172a (Figure 1J). Consequently, these cells were excluded from further analysis. A distinct population of cells also negative for CD14 and with a CD16 MFI of greater than 10 000 (CD14^−^CD16^++^; Figure 2C, panel Q4) was evident at relatively low proportion (0.9 ± 0.5%).

A major population of cells expressing CD14 was present at a relatively high proportion in PBMC (Figures 1G, 2B). Within the CD14^+^ population, cells with variable levels of CD16 were evident (Figure 2C, panels Q1 and Q2) similar to that previously reported by Ziegler-Heitbrock and Hofer [8] amongst others. Using the gating strategy suggested by these authors to be the most appropriate for monocyte populations, we demonstrated that the majority of the CD14^+^ cells expressed low levels of CD16 (16.2 ± 6.6% of the total PBMC; average CD16 MFI 366 ± 99; Figure 2C, panel Q1). A second population within the CD14^+^ cells which expressed higher levels of CD16 (average CD16 MFI 4039 ± 607; Figure 2C, panel Q2) was evident as a smaller and variable proportion in all animals studied (1.2 ± 0.4%).

**Figure 3:**
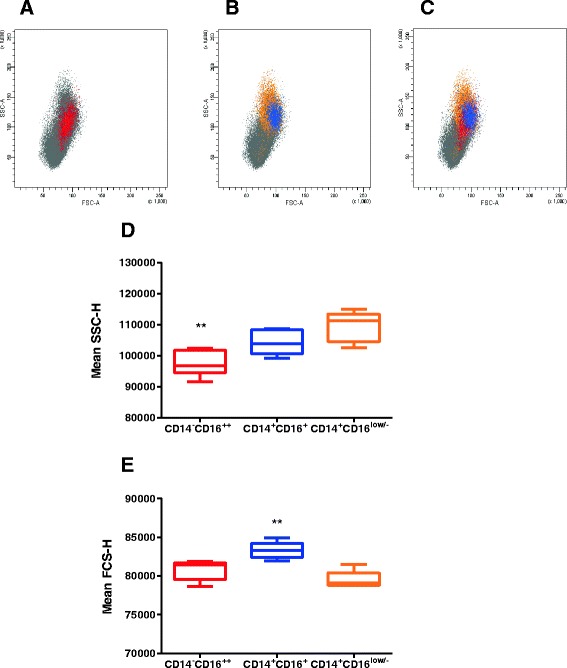
**Size and granularity of bovine myeloid sub-populations.** Live single PBMC were gated as shown in Figure 2C and each highlighted in different colours for identification in side and forward scatter plots. The size and granularity of the populations was determined by their characteristic side and forward scatter plots: in red, CD14^−^CD16^++^ (**A**); in orange, CD14^+^CD16^low/-^ and, superimposed, the CD14^+^CD16^+^ population in blue (**B**). Combining the plots (**C**) showed the difference of size and granularity of these cells. Data shown are for one representative animal out of six. (**D**, **E**) Box and whiskers plots for the arithmetic mean SSC-H (**D**) and FSC-H (**E**) for each myeloid subset (*n* = 6) are presented as the mean values ± SE, showing the difference in size and granularity of the populations. ** denotes significant difference (*p* < 0.001).

The cell populations gated based on their expression of CD14 and CD16 (Figure 2C, panels Q1, Q2, Q4) were then assessed for size (FSC) and granularity (SSC) (Figure 3A-E). Whilst the populations overlapped in terms of their FSC and SSC, it was clear that the CD14^−^CD16^++^ population (Figure 2C, panel Q4, Figure 3A, C, D highlighted in red) was significantly less granular than either the CD14^+^CD16^+^ population (Figure 2C, panel Q2, Figure 3B, C, D highlighted in blue) or the major CD14^+^CD16^low/-^ population (Figure 2C, panel Q1, Figure 3B, C, D highlighted in orange) (*p* = 0.001; Figure 3D). By contrast, the mean cell size (FSC) of the CD14^+^CD16^+^ population was significantly greater than either of the other two populations (*p* < 0.001; Figure 3E).

Based on these data, we hypothesised that the CD14^−^CD16^++^, CD14^+^CD16^+^ and CD14^+^CD16^low/-^ populations could have similar phenotypic and functional characteristics to the human monocyte populations reported widely in the literature: namely classical, intermediate and non-classical monocyte populations [2]. These cells have not been extensively characterised in ruminants and consequently we focused our investigations on these three populations.

### Cell surface expression characteristics of CD14^+^CD16^low/-^, CD14^+^CD16^+^ and CD14^−^CD16^++^ populations

**Figure 4:**
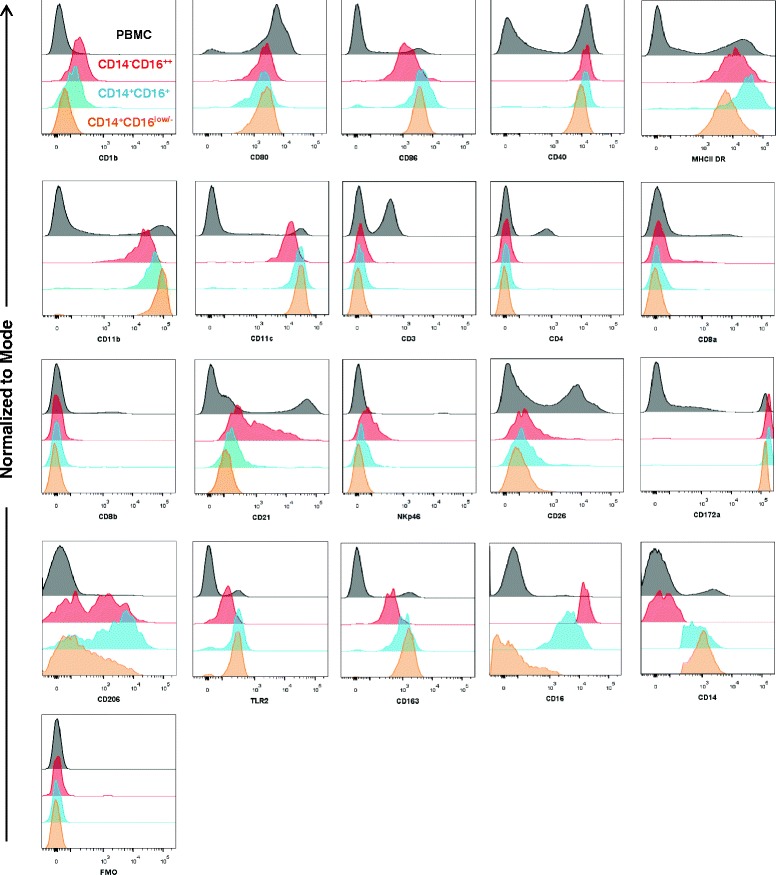
**Phenotypic profiles of bovine myeloid cell sub-populations.** Live gated PBMC were assessed for expression of CD16 and CD14 and a panel of molecules associated with antigen presentation, co-stimulation or specific cell lineages (see Table 1) by 3 colour flow cytometry. PBMC were stained with primary mAb to the specific molecules indicated and then with an isotype-specific PE conjugated secondary, followed by CD16 and CD14 conjugated to FITC or Alexa Fluor 647 respectively. Live, single PBMC were gated based on the expression of CD16 and CD14 as detailed in Figures 1 and 2C. Histograms show the levels of expression of selected markers in the cell populations studied, CD14^−^CD16^++^ (red), CD14^+^CD16^+^ (blue) and CD14^+^CD16^low/-^ (orange) compared to PBMC (black). Data shown are for one representative animal of four animals.

Three-colour flow cytometry was performed to investigate cell surface expression of a number of molecules in the CD14^−^CD16^++^ (Figure 2C, panel Q4, Figure 4 (red)); CD14^+^CD16^+^ (Figure 2C, panel Q2, Figure 3 (blue)) and CD14^+^CD16^low/-^ (Figure 2C, panel Q1, Figure 4 (orange)). Live, single cells were gated according to the expression of CD14/CD16 (Figure 2C) and the expression of a panel of molecules associated with specific cell lineages (lymphoid and myeloid) and specific functions (antigen presentation and co-stimulation) was assessed (Figure 4, Table 3). Representative flow cytometry histograms are shown in Figure 4, and the mean fluorescence intensity values of six individuals reported in Table 3. There were no significant differences between the three populations in the expression of CD172a, CD11c, CD40, MHCII-DR and CD80 which were uniformly high, whereas CD3, CD4, CD8α, CD8β, CD26 and NKp46 were consistently negative compared to FMO controls.

**Table 3:** **Phenotyping of CD14** ^**−**^ **CD16** ^**++**^ **, CD14** ^**+**^ **CD16** ^**+**^ **and CD14** ^**+**^ **CD16** ^**low/-**^ **cell populations**

**Cell marker**	**CD14** ^**−**^ **CD16** ^**++**^ **average MFI (SE)**	**CD14** ^**+**^ **CD16** ^**+**^ **average MFI (SE)**	**CD14** ^**+**^ **CD16** ^**low/-**^ **average MFI (SE)**	
CD1b	129 (28)^a^	113 (12)	54 (7)^b^	Antigen presentation Co-stimulatory molecules
CD80	2349 (378)	1590 (119)	1889 (66)	Antigen presentation Co-stimulatory molecules
CD86	1226 (172)^a^	2941 (291)	2403 (403)^b^	Antigen presentation Co-stimulatory molecules
CD40	9166 (1548)	7896 (1224)	6839 (967)	Antigen presentation Co-stimulatory molecules
MHC DR	46 014 (12 130)	63 800 (13650)	32 720 (9057)	Antigen presentation Co-stimulatory molecules
CD11b	20 755 (4334)^a^	32 631 (5299)	47 893 (9406)^b^	Antigen presentation Co-stimulatory molecules
CD11c	7575 (3295)	12 828 (4748)	21 093 (11339)	Antigen presentation Co-stimulatory molecules
CD3	<10^a^	43 (11)^b^	36 (8)^b^	Lymphoid cell markers
CD4	<10	<10	<10	Lymphoid cell markers
CD8α	<10	28 (3)	14 (4)	Lymphoid cell markers
CD8β	<10	10 (5)	<10	Lymphoid cell markers
CD21	618 (140)^A^	247 (23)^B^	136 (18)^B^	Lymphoid cell markers
NKp46	38 (16)	34 (3)	17 (2)	Lymphoid cell markers
CD26	147 (37)	151 (22)	110 (21)	Lymphoid cell markers
CD172a	96 077 (31018)	100 076 (31620)	89 046 (28726)	Myeloid cell markers
CD206	649 (260)	1533 (389)^a^	191 (32)^b^	Myeloid cell markers
TLR2	92 (25)	299 (41)	261 (96)	Myeloid cell markers
CD163	409 (68)^A^	1273 (58)^B^	1898 (222)^C^	Myeloid cell markers
CD16	18 344 (1448)^A^	4039 (607)^B^	366 (99)^C^	Myeloid cell markers
CD14	20 (16)^A^	978 (78)^B^	1748 (337)^B^	Myeloid cell markers

Three colour flow cytometry was carried out on PBMC stained with selected mAb (details in Table 1) as primary antibodies and stained with an isotype-specific R:PE conjugated secondary mAb, followed by directly conjugated mAbs CD16 and CD14. Following live/dead and singlets gating, PBMC were then gated based on the expression of CD16 and CD14 as CD14^-^CD16^++^, CD14^+^CD16^+^ and CD14^+^ CD16^low/-^ as shown in Figure 2 and expression of the markers was then analysed. Geometric mean fluorescence intensity (MFI), corrected with the MFI of its corresponding FMO, for each of the molecules is shown in the table as an arithmetic mean and standard error -SE (*n* = 4). Different letters denote significant difference in marker expression levels (MFI) between the three myeloid populations (*p* < 0.05 (lower case) and *p* < 0.001 (upper case)).

For a number of molecules assessed, there were significant differences between the populations (Table 3); notably between the CD14^+^CD16^low/-^ (Figure 4 (orange)) and the CD14^−^CD16^++^ (Figure 4 (red)) populations. Significantly higher expression of CD1b was observed on the CD14^−^CD16^++^ cells compared with the CD14^+^CD16^low/-^ cells (*p* = 0.039). For CD86 and CD11b significant differences between the CD14^+^CD16^low/-^ cells and the CD14^−^CD16^++^ cells were also observed (*p* = 0.009 and 0.04 respectively). Although the majority of the CD14^−^CD16^++^ cells were negative for CD21 (70 ± 13.2%), a proportion of cells (29.9 ± 13.4%) exhibited a broad range of CD21 expression, albeit lower than the expression observed on B cells in PBMC (Figure 4 (compare red with grey histograms)). The overall MFI (618 ± 140) was significantly higher than that for CD21 on both CD14^+^ populations (*p* = 0.001). All three myeloid cell populations expressed CD206, with staining patterns that indicated that varying proportions of two subsets, one CD206 positive and one CD206 negative, may be present in each population. In particular the majority of CD14^+^CD16^+^ cells expressed CD206 at a significantly higher level compared to the CD14^+^CD16^low/-^ population (*p* = 0.002). Expression of CD163 was significantly different across the three populations with the lowest expression observed on the CD14^−^CD16^++^ population (*p* < 0.001, when compared to each CD14^+^ population). A trend towards lower expression of TLR2 by the CD14^−^CD16^++^ cells was also observed (*p* = 0.091).

### Cytokine levels secreted by CD14^+^CD16^low/-^, CD14^+^CD16^+^ and CD14^−^CD16^++^ populations in the presence and absence of LPS stimulation

**Figure 5:**
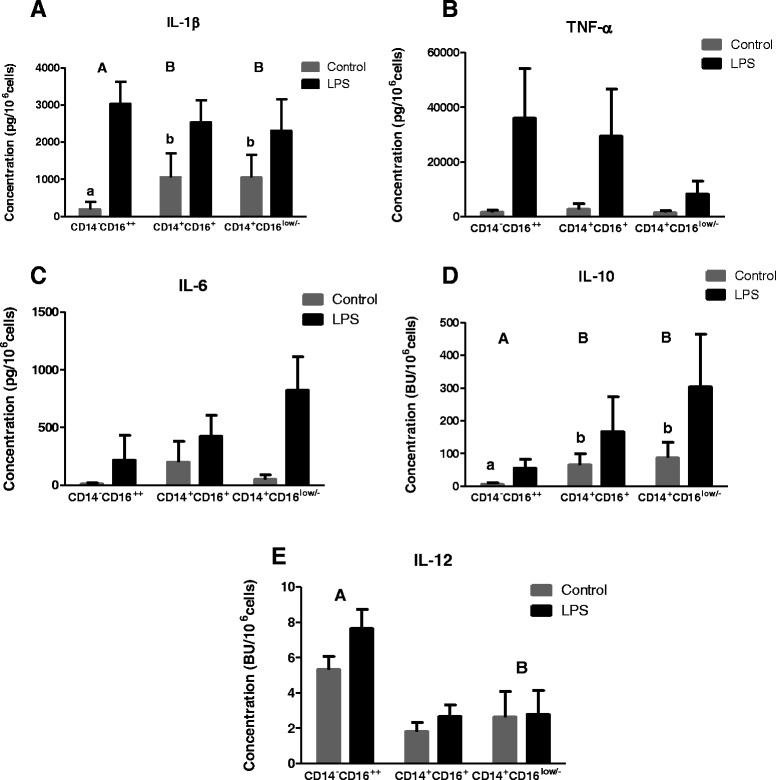
**LPS stimulation of distinct myeloid cell populations induces differential cytokine secretion profiles.** Cells were purified by using gating strategy shown in Figure 2C and stimulated for 18 h with LPS (black bars) or medium alone (grey bars). The levels IL-1b (A), TNF-a (B), IL-6 (C), IL-10 (D) and IL-12 (E) secreted into culture supernatants were measured by ELISA. The data is expressed as the concentration of cytokine in picograms (pg) or biological activity (biological units (BU)) secreted by 10^6^ cells. Different letters denote statistical significance between the different cell populations: capital letters represent significant difference of the fold increase induced by stimulation (stimulated value divided by un-stimulated control), while lowercase denotes significance in the comparison of un-stimulated values. Results are shown as the mean values with error bars indicating ± SE of the sorted populations from six animals.

To further investigate the differences of these myeloid cell populations and assess their functional characteristics, the three cell subpopulations identified in Figure 2C (CD14^−^CD16^++^; CD14^+^CD16^+^ and CD14^+^CD16^low/-^) were purified from PBMC of six animals. The purity and the number of cells obtained were determined after sorting (Table 4). The cytokine expression profile of each population was assessed 18 h following stimulation with LPS and compared to that of unstimulated cells (Figure 5A-E). Although considerable variation in cytokine levels was found between animals, there were significant differences between the three myeloid subsets in terms of the fold-change in response to LPS compared to the controls for IL-1β (*p* < 0.001), IL-10 (*p* = 0.022) and IL-12 (*p* = 0.018) (Figure 5A, D and E). Greater variation between animals in the constitutive levels of IL-1β was observed in the two CD14^+^ populations whereas the CD14^−^CD16^++^ cells consistently had negligible levels of this cytokine (Figure 5A), which were significantly lower than that produced constitutively by either of the CD14^+^ populations (*p* = 0.001). However, the CD14^−^CD16^++^ cells did respond to LPS by secreting significantly more IL-1β compared to the medium only controls (*p* < 0.001). No significant difference between the three cell populations was found in the production of TNF-α or IL-6 (Figure 5B and C).

**Table 4:** **Myeloid cell purification**

**Animal ID**	**CD14** ^**−**^ **CD16** ^**++**^	**CD14** ^**+**^ **CD16** ^**+**^	**CD14** ^**+**^ **CD16** ^**low/-**^
**201243**	400 000	1 100 000	1 100 000
**601240**	350 000	2 400 000	2 800 000
**501246**	500 000	1 800 000	3 500 000
**301244**	380 000	1 200 000	3 000 000
**301237**	550 000	1 650 000	3 500 000
**101249**	650 000	730 000	1 500 000
**Example of Purity**	96.5%	88.5%	90.6%

Numbers of cells collected in each of the cell sorts for cytokine response to LPS stimulations of myeloid populations. Purities were consistently above 85%; a representative example of the purities obtained is shown.

As observed for IL-1β, the CD14^−^CD16^++^ cells secreted significantly lower constitutive levels of IL-10 compared to the other two myeloid subsets which showed greater variation between animals following LPS stimulation (*p* = 0.037, Figure 5D). Low levels of IL-12 were secreted by all 3 myeloid subpopulations. However the CD14^−^CD16^++^ population exhibited a significantly greater fold-change in response to LPS compared to the controls than the major CD14^+^CD16^low/-^ population (*p* = 0.018, Figure 5E).

### CD14^−^CD16^++^ cells induce higher allogeneic MLR than either of the CD14^+^ populations

**Figure 6:**
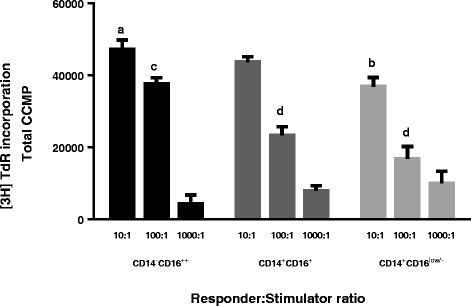
**Distinct myeloid cell populations induce allogeneic mixed leukocyte reactions of differing intensity.** Three cell populations (CD14^−^CD16^++^, CD14^+^CD16^+^, CD14^+^CD16^low/-^) were purified as shown in Figure 2C, irradiated and cultured at the indicated cell ratios with responder PBMC (10^5^ per well) for 5 days. Proliferation was measured by the incorporation of methyl-3H thymidine ([3H]TdR; 0.5 μCi per well) for the final 18 h of culture. Data are presented as the corrected counts per minute (ccpm) averaged over 3 min. Error bars denote ± SE of the technical replicates and letters represent statistical significance between proliferation induced by each cell population for each responder:stimulator ratio for *p* < 0.05. One representative experiment of three is shown.

To further investigate the nature of the CD14^−^CD16^++^, CD14^+^CD16^+^ and CD14^+^CD16^low/-^ cell populations, their capacity to induce lymphocyte proliferation was assessed in an allogeneic MLR (Figure 6). At the highest responder:stimulator ratio (10:1), the CD14^−^CD16^++^ population showed a significantly greater capacity to induce proliferation when compared to the CD14^+^CD16^low/-^ cells (*p* = 0.029). A more pronounced difference was observed when the ratio of responders to stimulators was 100:1, where the proliferation induced by the CD14^−^CD16^++^ cells was significantly higher than that induced by either of the CD14^+^ populations (*p* < 0.001).

Since few significant differences were observed across a number of parameters (antigen presentation, cytokine secretion, cell surface phenotype) between the CD14^+^CD16^low/-^ cells and the CD16^+^CD14^+^cells, additional analyses were focused on the comparison of the total CD14^+^ population to the CD14^−^CD16^++^ cells, as described below.

### Gene expression levels

**Figure 7:**
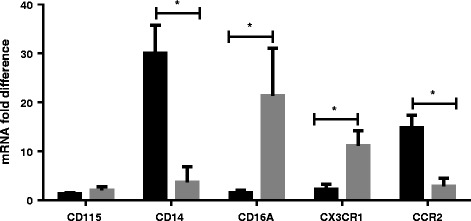
**Summary of the RT-qPCR analysis of the transcriptional profile of monocyte subsets.** Arithmetic mean of CD115, CD14, CD16A (FCGR3A), CCR2 and CX3CR1 mRNA fold differences detected in CD14^+^ (black bars) and CD16^++^ (grey bars) cells. The results are expressed as the fold difference compared to the sample with the lowest expression for each gene. Error bars illustrate the standard deviation of four animals/biological replicates. * denotes that the variation in expression was statistically significantly different by *t* test (*p* < 0.05).

To assess expression of key molecules reported to be associated with monocyte populations we measured expression of a panel of genes in purified monocyte populations from four animals by RT-qPCR. This analysis confirmed that the receptor for colony stimulating factor 1 (CSF1R; CD115) was expressed at similar levels in both CD14^+^ and CD14^−^CD16^++^ samples (Figure 7), although there was more animal to animal variation within the CD14^−^CD16^++^ samples. CD115 was expressed by both CD14^+^CD16^low/-^ and CD14^+^CD16^+^ populations, but not by NK cells (results not shown). CD14 mRNA levels were significantly higher, on average 8.2 fold (*p* = 0.001), in CD14^+^ cells and conversely CD16A (FCGR3A) levels were significantly higher, on average 14.1 fold (*p* = 0.027), in CD14^−^CD16^++^ cells. Again there was considerable variation in the expression of CD16A in the CD14^−^CD16^++^ cells (Figure 7). Further analysis of genes known to be differentially expressed in human and murine monocyte subsets revealed that CX3CR1 was expressed at significantly higher levels in CD14^−^CD16^++^ cells, with the expression being on average 4.9 fold higher than in CD14^+^ cells (*p* = 0.012). In contrast, another chemokine receptor, CCR2, was expressed at significantly higher levels, on average 5.3 fold, in CD14^+^ cells than CD14^−^CD16^++^ cells (*p* = 0.004). The expression of CD163 was also investigated and transcripts were detected in CD14^+^ cells, but not in CD14^−^CD16^++^ cells (data not shown). Therefore, the phenotype of the bovine monocyte populations are: CD14^+^CD16^+/−^CD163^+^CCR2^+^ and CD14^−^CD16^++^CX3CR1^+^.

### CD14^−^CD16^++^ cells have a significantly higher endocytic capacity compared to CD14^+^ cells

**Figure 8:**
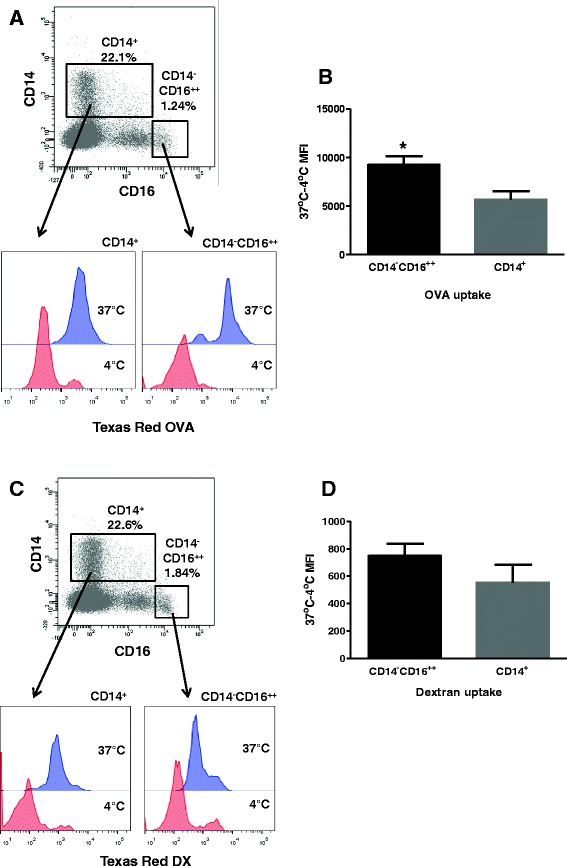
**Differential endocytosis of OVA and Dextran by subpopulations of myeloid cells**. PBMC were isolated and incubated for 30 min with 100 μg/mL of TexasRed-OVA (**A**, **B**) or TexasRed-Dextran (**C**, **D**) at 37 °C or on ice (to assess background uptake). The cells were subsequently washed and stained with conjugated CD14 and CD16 antibodies and analysed by flow cytometry. Live, single cells were gated as in Figure 1 and the MFI was calculated for the CD14^−^CD16^++^ and CD14^+^ gated populations (**A**, **C**). The results shown (**B**, **D**) are mean values with error bars showing ± SE for four animals and represent the TexasRed MFI at 37 °C minus MFI on ice (4 °C).

To assess the capacity for antigen uptake by the CD14^+^ and CD14^−^CD16^++^ cell populations, PBMC from four animals were incubated with either Dextran (DX), a complex glucan taken up via the mannose receptor (MR; CD206) by macropinocytosis [43] or Ovalbumin (OVA) a protein taken up by clathrin-coated pits [44], both fluorescently labelled with TexasRed (TR). The cell populations were then identified within PBMC as CD14^+^ or CD14^−^CD16^++^ after incubation with CD14 and CD16 mAbs. Low level internalisation was observed when cells were incubated on ice for both TR-OVA (Figure 8A) and TR-DX (Figure 8C); while significantly higher levels of uptake were observed after incubation at 37 °C. Comparing uptake between the cell populations revealed that the CD14^−^CD16^++^ cells internalised significantly higher levels of TR-OVA when compared to the CD14^+^ cells (*p* = 0.03, Figure 8B).

Both cell types were equally able to endocytose TR-DX and although the CD14^−^CD16^++^ cells showed a trend towards higher internalisation, when the TR MFI was compared to that of the CD14^+^ cells (Figure 8D) this difference did not reach statistical significance.

### Distinct populations of myeloid cells based on differential expression of CD14 and CD16 are also evident in sheep

**Figure 9:**
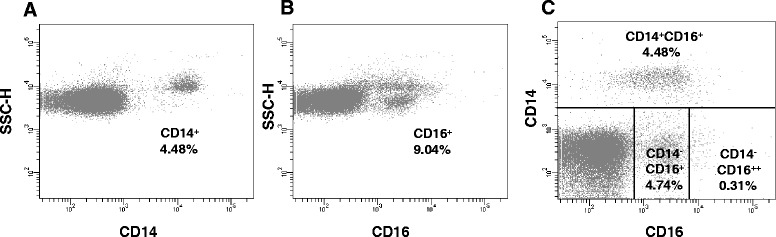
**Subpopulations of myeloid cells with differential expression of CD14 and CD16 are present in ovine peripheral blood.** The expression of CD16 and CD14 was determined by single and two colour flow cytometry in freshly isolated ovine PBMC. Live, single cells gated as in Figure 1 were further analysed for the expression of CD14 (**A**), CD16 (**B**) or were double stained with mAbs to CD16 and CD14 (**C**). Distinct populations of cells were identified (C): CD14^+^CD16^low/+^, CD14^−^CD16^++^ and CD14^−^CD16^+^. Data shown are for one representative animal out of eight.

The expression of CD14 and CD16 by ovine PBMC was investigated in eight animals and compared to that of bovine peripheral blood. As in cattle, single staining with CD14 revealed one distinct population. However, the overall percentage of cells expressing CD14 in ovine peripheral blood was significantly lower when compared to bovine blood, with only 4.1 ± 0.9% of the cells expressing CD14 (Figure 9A). On the other hand, ovine blood contained a comparable number of cells expressing CD16 (10.6 ± 5.1% of the total PBMC Figure 9B) to bovine blood. Within the CD14^−^ fraction, CD16 was expressed at a moderate level by the majority of cells (Figure 9C) as previously described by others [28]. Very few CD14^−^CD16^++^ cells were evident in ovine blood (0.6 ± 0.4% of the total PBMC, CD16 MFI of 447 ± 25) whilst only a single CD14^+^ population (4.1 ± 0.9%) was discernible with variable CD16 expression (CD16 MFI of 50 ± 5). These low percentages precluded detailed investigation of the phenotype and function of these different myeloid populations in ovine peripheral blood.

## Discussion

In this study we focused our investigations on the identification of monocyte subsets present in the peripheral blood of cattle and sheep using two markers commonly used to identify monocyte populations in human, namely CD14 and CD16. In agreement with previous reports in ruminants [28,29,45-47], single staining with mAbs specific for these molecules revealed one distinct population based on levels of CD14 expression, whereas CD16^+^ cells displayed a range of expression levels. We have shown that two clearly distinct CD172a^+^ cell populations are present in bovine PBMC consisting of a major subset of CD14^+^CD16^low/-^ cells and a minor population of CD14^−^CD16^++^ cells (88.1 ± 2.9%, and 5 ± 1.7% respectively of CD172a^+^ cells). These can be distinguished from NK cells by their lack of NKp46 expression and higher CD16 expression. As in humans [8] there was also present a more diffuse minor population (6.8 ± 2.0% of CD172a^+^ cells) expressing a spectrum of CD14 and CD16 levels in between those expressed by the more distinct monocyte subsets. In this regard, our data essentially agree with that recently published by Hussen et al. [31]. Although sheep are a closely related species, diverging from cattle around 31 million years ago [48], there were fewer circulating ovine monocytes and these exhibited very different CD14 and CD16 staining patterns from bovine monocytes. A single major CD14^+^CD16^+^ population was discerned with few CD14^−^CD16^++^ cells. It is unlikely that these differences were simply related to different antibody affinities for ovine CD14 or CD16 as similar results have been obtained with mAb specific for ovine CD14 (unpublished observations). Furthermore, ovine NK cells had similar levels of CD16 expression to bovine NK cells. It is possible that these differences in monocyte subsets in the circulation are not absolute, but reflective of differences in the steady state migration to and from the blood, as well as proliferative capacity and cell death rates of bovine and ovine monocytes. However these differences warrant further investigation as they may, at least partly, explain different responses of cattle and sheep to a range of pathogens.

The three bovine monocyte populations expressed CD115, confirming their monocyte lineage and providing further evidence that CD115 is the canonical conserved marker of monocytes in vertebrate species. Although some progress has been made on the generation of anti-CD115 mAb for species other than humans and mice, including chickens and pigs [49,50], so far no anti-CD115 mAb for ruminants or other species has been reported to enable confirmation of the level of expression of CD115 at the cell surface. The CD172a^+^CD14^−^CD16^++^ population was the smallest in size, the least granular and, expressed little or no CD14 and CD163 and relatively low levels of CCR2 and CD11b, but was shown to express high levels of CX3CR1. These cells thus resemble the human non-classical population of monocytes [8] and the mouse Ly6C^lo^ monocyte population [51]. The CD14^+^ population, on the other hand, expressed the highest levels of CD14, the lowest levels of CD16, over four times the level of CD163 expressed by the CD14^−^CD16^++^ cells (Table 3), higher levels of CCR2 and lower levels of CX3CR1. This major monocyte population thus has a similar phenotype to the human classical population [52], mouse Ly6C^hi^ monocytes [51] and pig CD14^+^ classical population [18]. The differential expression of the chemokine receptors indicates that the two monocyte populations are likely to migrate differently from the blood into the tissues in response to inflammatory signals as well as under steady-state conditions [5,10]. In contrast to human and mouse monocytes, or indeed the previous report on bovine monocytes [31], we were able to show that bovine classical CD14^+^ monocytes were similar in size to the non-classical population. The reason for the latter discrepancy is unclear, although porcine monocytes have been reported to increase in size following differentiation in the presence of CSF1 [18]. Instead, the intermediate CD14^+^CD16^+^ population consisted of the largest cells which is in contrast to Hussen et al. [31] which may reflect our different gating strategies and exclusion of NK cells from our analysis.

The current nomenclature for human blood monocytes, which segregates them into three subsets [7,11], has been recognised to be subjective as different antibody clones and gating strategies have been used, and consequently the populations studied in different reports may also be different [8]. These studies have generated some conflicting data, with some authors suggesting that the classical and intermediate monocytes are clustered separately from the non-classical monocytes [53], whilst others state that it is actually the intermediate and the non-classical which are more closely related [2]. Thus, as described by Ziegler-Heitbrock and Hofer [8], gating strategies for monocytes are essential particularly when studying the intermediate monocyte population. Given this, we ensured our gating strategy was consistent across different animals and took account of threshold for CD14 and CD16 negative populations, as well as the intermediate CD16 levels expressed by some monocytes and NK cells. This enabled us to clearly distinguish between the major classical monocyte CD14^+^CD16^low/-^ subset and the non-classical CD14^−^CD16^++^ subset. In addition, we defined the CD14^−^CD16^++^ monocyte population on the basis of lack of CD14 expression and distinctly higher expression of CD16 than the NK cell population. This is in contrast to the previous report which used a different gating strategy and defined CD14^+^CD16^+^ cells as the bovine non-classical monocyte subset [31].

Even though the distinction of the intermediate population from the CD14^+^CD16^low/-^ and the CD14^+^CD16^+^ cells was less clear, we applied a consistent gate across all samples and used this strategy together with cell sorting to purify the three populations for functional analysis. The CD14^+^CD16^+^ population mainly expressed levels of cell surface molecules that varied between the levels in the other two monocyte populations, and are thus similar to the intermediate monocyte phenotype described in humans and mice [2,54]. This would be in line with the view that these cells are maturing from CD14^+^CD16^low/-^ monocytes to become CD14^−^CD16^++^ monocytes [2]. In addition, all monocyte subsets were negative for lymphoid markers, with one exception (see below).

In order to explore the nature of the three CD115^+^CD172a^+^ monocyte populations further, three colour flow cytometry was carried out with selected molecules. The three populations were positive for most of the APC markers, co-stimulatory molecules and myeloid markers. In terms of antigen presenting function all three populations expressed similar levels of CD40, CD80 and bovine MHC class II DR, although the levels on intermediate CD14^+^CD16^+^ cells showed a trend towards higher expression. This would be in agreement with the earlier report on monocyte populations in cattle [31] and also with human and porcine intermediate monocytes [2,18]. The CD14^+^CD16^low/-^ population expressed higher levels of CD86 than the CD14^−^CD16^++^ population whereas the CD14^−^CD16^++^ expressed higher levels of CD1b. CD1 is a multi-gene family in cattle and different CD1 molecules are expressed on B cells and DC, although B cells do not always express the proteins on the cell surface [55]. This molecule is involved in presentation of lipid antigens and is important in Mycobacterial infections [56]. The antibody used in our study has been shown to recognise CD1b3 which is expressed on cattle afferent lymph dendritic cells (ALDC) and immature DC derived from monocytes [55]. It is likely that the relatively low levels of CD14^−^CD16^++^ cells in peripheral blood together with the low, though significant, cell surface expression of CD1b3, has precluded its detection previously in PBMC. Intriguingly a subset of CD14^−^CD16^++^ were also positive for CD21, a marker typically present on B cells, which has however also been reported to be expressed by a subpopulation of bovine ALDC [57] and in human follicular dendritic cells [58]. Further, both the intermediate and CD14^−^CD16^++^ had subsets expressing CD206 or the mannose receptor, a C-type lectin typically present in macrophages and immature dendritic cells [59]. This receptor is active in endocytosis and phagocytosis and recognises specific mannosylated protein antigens found on the surface of pathogens, playing a key role in both the innate and adaptive immune systems [60]. However the higher expression of CD206 on CD14^−^CD16^++^ monocytes did not result in significantly higher uptake of dextran, although they did take up higher levels of ovalbumin, a process which can also be dependent on CD206 [61]. Thus it is possible that the same subset of the CD14^−^CD16^++^ monocytes express CD172a, CD1b3, CD21 and CD206. If so, these cells would have a similar phenotype to the subpopulation of bovine CD172a^+^CD1b3^+^CD21^+^CD206^+^ ALDC which have greater capacity to take up both dextran and ovalbumin than other ALDC populations [59]. This specific subpopulation of ALDC also phagocytose pathogens such as *M. bovis* but are less effective at antigen presentation to T cells [59]. Further multi-colour cytometric analysis is required to confirm whether there is a subpopulation of CD172a^+^CD14^−^CD16^++^CD1b3^+^ monocytes which also express CD21 and CD206. Certainly, further monocyte subpopulations that are not conventionally defined by CD14 and CD16 have been reported in humans, including Tie^+^ monocytes that overlap with the intermediate monocyte subset and 6-sulpho LacNAc^+^ (SLAN; a carbohydrate modification of P-selectin glycoprotein ligand 1) monocytes that appear to be a subset of CD14^−^CD16^++^ monocytes [11].

Although data are now beginning to appear about monocyte subsets in other mammalian species [52], information is very limited in species within the artiodactyla clade, partly due to the paucity of antibodies against specific particular markers, and can have conflicting conclusions. Porcine monocytes for example, differ in expression of surface CD14 and the scavenger receptor for the hemoglobin-haptoglobin complex, CD163. In early reports the porcine population identified as CD163^+^CD14^−^, was related to the human CD14^+^CD16^+^ population due to the functional and phenotypic characteristics of these cells [62,63]. Recently, this comparison has been challenged and comparing differential gene expression data of monocyte populations from human, mouse and pig, no obvious equivalent to human non-classical monocytes could be found. Despite this, a relationship between porcine CD163^low^CD14^++^ cells and the human classical CD14^++^ population was established [18]. In contrast to pigs, but similar to humans [64,65], CD163 was weakly positive in the bovine CD14^−^CD16^++^ cells compared to the higher level present in the bovine CD14^+^CD16^low/-^ monocytes and this agrees with the previous report [31]. In horses, limited analysis suggests that the majority of CD14^+^ monocytes are CD16^−^, and a minority are CD16^+^, possibly corresponding to the classical and intermediate monocytes of other species [66] but so far there has been no further phenotyping or functional analysis of equine monocyte subsets.

To investigate the three monocyte populations further, and to establish if these cells differ in function, we separated the populations based on their differential expression of CD14 and CD16 using fluorescence-activated cell sorting. A previous report had suggested that CD14^+^CD16^+^ monocytes did not up-regulate mRNA for CXCL8 or IL-1β in response to LPS, suggesting that the cells they referred to as non-classical were not inflammatory [31]. In contrast, in our hands, all three populations responded to LPS by producing IL-1β protein including the CD14^−^CD16^++^ cells, which had the lowest constitutive levels of IL-1β. The contradictory results may reflect differences in the gating strategies and selection methods, as we sorted the cell populations using flow cytometry to high purity enabling exclusion of CD16^+^ NK cells. Unfortunately considerable variation between animals precluded any conclusions with respect to the pro-inflammatory cytokines TNF-α and IL-6 and warrants further investigation with a larger sample set as well as other agonists.

Overall, it appears that the classical CD14^+^CD16^low/-^ and intermediate monocytes produce both constitutively more IL-10 and the least IL-12, in comparison with the CD14^−^CD16^++^. Secretion of IL-10 is a feature of human, mouse and pig classical monocytes whereas non-classical monocytes produce little or no IL-10 [2,8,67]. In contrast some reports suggest that non-classical monocytes, in particular the human CD14^−^CD16^++^SLAN^+^ monocyte subset [68], have a propensity to produce IL-12 in response to stimulation [42]. Unfortunately we were unable to obtain an anti-SLAN mAb which cross-reacted with bovine cells (results not shown).

Finally, we investigated the capacity of all three populations to induce an allogeneic MLR using the three populations as stimulators and PBMC as responders from two animals with different homozygous MHC class I haplotypes. The CD14^−^CD16^++^ monocytes had a significantly higher capacity to induce proliferation in PBMC than either of the CD14^+^ subsets. Nonetheless it remains unclear what features of this population enable it to induce an allogeneic response, as the majority of antigen presenting and co-stimulatory molecules are not expressed at higher levels in these cells compared to the CD14^+^ subsets. Further phenotypic and functional work on the CD14^−^CD16^++^ monocytes and potential subsets within this classification may provide additional insights.

In summary, we have extended earlier findings [31], with additional phenotypic and functional characterisation of bovine monocytes and added information on ovine monocyte subsets. In many ways the major CD14^+^ population in cattle blood shares many features with classical monocytes of other species. Although we carefully defined a consistent gating strategy, it is not clear that the less distinct CD14^+^CD16^+^ cells are part of a separate population but could represent the maturation and differentiation of the major CD14^+^ monocytes into CD14^−^CD16^++^ monocytes. This latter subset shares many features with non-classical monocytes of other species and also may contain further subset(s) which share features more commonly associated with DC. The presence and function of all three populations during infections and other inflammatory diseases in cattle needs to be explored further to determine what role they might play in immunity, and whether their levels in blood might be used as correlates of protection and pathogenesis in ruminants.

## References

[1] Serbina NV, Jia T, Hohl TM, Pamer EG (2008). Monocyte-mediated defense against microbial pathogens. Annu Rev Immunol.

[2] Wong KL, Tai JJ-Y, Wong W-C, Han H, Sem X, Yeap W-H, Kourilsky P, Wong S-C (2011). Gene expression profiling reveals the defining features of the classical, intermediate, and nonclassical human monocyte subsets. Blood.

[3] Ziegler-Heitbrock HW, Fingerle G, Ströbel M, Schraut W, Stelter F, Schütt C, Passlick B, Pforte A (1993). The novel subset of CD14+/CD16+ blood monocytes exhibits features of tissue macrophages. Eur J Immunol.

[4] Ziegler-Heitbrock L (2007). The CD14+ CD16+ blood monocytes: their role in infection and inflammation. J Leukoc Biol.

[5] Auffray C, Fogg DK, Narni-Mancinelli E, Senechal B, Trouillet C, Saederup N, Leemput J, Bigot K, Campisi L, Abitbol M, Molina T, Charo I, Hume DA, Cumano A, Lauvau G, Geissmann F (2009). CX3CR1+ CD115+ CD135+ common macrophage/DC precursors and the role of CX3CR1 in their response to inflammation. J Exp Med.

[6] Segura E, Villadangos JA (2009). Antigen presentation by dendritic cells in vivo. Curr Opin Immunol.

[7] Ziegler-Heitbrock L, Ancuta P, Crowe S, Dalod M, Grau V, Hart DN, Leenen PJM, Liu Y-J, MacPherson G, Randolph GJ, Scherberich J, Schmitz J, Shortman K, Sozzani S, Strobl H, Zembala M, Austyn JM, Lutz MB (2010). Nomenclature of monocytes and dendritic cells in blood. Blood.

[8] Ziegler-Heitbrock L, Hofer TP (2013). Towards a refined definition of monocyte subsets. Front Immunol.

[9] Ziegler-Heitbrock HW (1996). Heterogeneity of human blood monocytes: the CD14 + CD16+ subpopulation. Immunol Today.

[10] Geissmann F, Jung S, Littman D (2003). Blood monocytes consist of two principal subsets with distinct migratory properties. Immunity.

[11] Wong K, Yeap W, Tai J, Ong S, Dang T, Wong S (2012). The three human monocyte subsets: implications for health and disease. Immunol Res.

[12] Frankenberger M, Sternsdorf T, Pechumer H, Pforte A, Ziegler-Heitbrock H (1996). Differential cytokine expression in human blood monocyte subpopulations: a polymerase chain reaction analysis. Blood.

[13] Belge K, Dayyani F, Horelt A, Siedlar M, Frankenberger M, Frankenberger B, Espevik T, Ziegler-Heitbrock L (2002). The proinflammatory CD14(+)CD16(+)DR(++) monocytes are a major source of TNF. J Immunol.

[14] Seok J, Warren HS, Cuenca AG, Mindrinos MN, Baker HV, Xu W, Richards DR, McDonald-Smith GP, Gao H, Hennessy L, Finnerty CC, López CM, Honari S, Moore EE, Minei JP, Cuschieri J, Bankey PE, Johnson JL, Sperry J, Nathens AB, Billiar TR, West MA, Jeschke MG, Klein MB, Gamelli RL, Gibran NS, Brownstein BH, Miller-Graziano C, Calvano SE, Mason PH (2013). Genomic responses in mouse models poorly mimic human inflammatory diseases. Proc Natl Acad Sci U S A.

[15] Bem RA, Domachowske JB, Rosenberg HF (2011). Animal models of human respiratory syncytial virus disease. Am J Physiol Lung Cell Mol Physiol.

[16] Entrican G, Wattegedera SR, Griffiths DJ (2015). Exploiting ovine immunology to improve the relevance of biomedical models. Mol Immunol.

[17] Jorgensen F, Hobolth A, Hornshoj H, Bendixen C, Fredholm M, Schierup M (2005). Comparative analysis of protein coding sequences from human, mouse and the domesticated pig. BMC Biol.

[18] Fairbairn L, Kapetanovic R, Beraldi D, Sester DP, Tuggle CK, Archibald AL, Hume DA (2013). Comparative analysis of monocyte subsets in the pig. J Immunol.

[19] Buddle BM, Skinner MA, Neil Wedlock D, de Lisle GW, Martin Vordermeier H, Glyn Hewinson R (2005). Cattle as a model for development of vaccines against human tuberculosis. Tuberculosis.

[20] Waters WR, Palmer MV, Thacker TC, Davis WC, Sreevatsan S, Coussens P, Meade KG, Hope JC, Estes DM (2011). Tuberculosis immunity: opportunities from studies with cattle. Clin Dev Immunol.

[21] Bean AGD, Baker ML, Stewart CR, Cowled C, Deffrasnes C, Wang L-F, Lowenthal JW (2013). Studying immunity to zoonotic diseases in the natural host - keeping it real. Nat Rev Immunol.

[22] Jann O, Werling D, Chang J-S, Haig D, Glass E (2008). Molecular evolution of bovine Toll-like receptor 2 suggests substitutions of functional relevance. BMC Evol Biol.

[23] Smith S, Jann O, Haig D, Russell G, Werling D, Glass E, Emes R (2012). Adaptive evolution of Toll-like receptor 5 in domesticated mammals. BMC Evol Biol.

[24] Entrican G, Wheelhouse N, Wattegedera SR, Longbottom D (2012). New challenges for vaccination to prevent chlamydial abortion in sheep. Com Immunol Microbiol Infect Dis.

[25] Arsenault RJ, Li Y, Bell K, Doig K, Potter A, Griebel PJ, Kusalik A, Napper S (2012). Mycobacterium avium subsp. paratuberculosis inhibits gamma interferon-induced signaling in bovine monocytes: insights into the cellular mechanisms of Johne’s disease. Infect Immun.

[26] Sacco RE, McGill JL, Pillatzki AE, Palmer MV, Ackermann MR (2013). Respiratory syncytial virus infection in cattle. Vet Pathol.

[27] Storset AK, Kulberg S, Berg I, Boysen P, Hope JC, Dissen E (2004). NKp46 defines a subset of bovine leukocytes with natural killer cell characteristics. Eur J Immunol.

[28] Elhmouzi-Younes J, Boysen P, Pende D, Storset AK, Le Vern Y, Laurent F, Drouet F (2010). Ovine CD16+/CD14- blood lymphocytes present all the major characteristics of natural killer cells. Vet Res.

[29] Sopp P, Kwong LS, Howard CJ (1996). Identification of bovine CD14. Vet Immunol Immunopathol.

[30] Berthon P, Hopkins J (1996). Ruminant cluster CD14. Vet Immunol Immunopathol.

[31] Hussen J, Düvel A, Sandra O, Smith D, Sheldon IM, Zieger P, Schuberth H-J (2013). Phenotypic and functional heterogeneity of bovine blood monocytes. PLoS One.

[32] Ellis SA, Holmes EC, Staines KA, Smith KB, Stear MJ, McKeever DJ, MacHugh ND, Morrison WI (1999). Variation in the number of expressed MHC genes in different cattle class I haplotypes. Immunogenetics.

[33] Gaddum RM, Cook RS, Furze JM, Ellis SA, Taylor G (2003). Recognition of bovine respiratory syncytial virus proteins by bovine CD8+ T lymphocytes. Immunology.

[34] Kwong LS, Hope JC, Thom ML, Sopp P, Duggan S, Bembridge GP, Howard CJ (2002). Development of an ELISA for bovine IL-10. Vet Immunol Immunopathol.

[35] Hope JC, Kwong LS, Entrican G, Wattegedera S, Vordermeier HM, Sopp P, Howard CJ (2002). Development of detection methods for ruminant interleukin (IL)-12. J Immunol Methods.

[36] Kwong LS, Thom M, Sopp P, Rocchi M, Wattegedera S, Entrican G, Hope JC (2010). Production and characterization of two monoclonal antibodies to bovine tumour necrosis factor alpha (TNF-α) and their cross-reactivity with ovine TNF-α. Vet Immunol Immunopathol.

[37] Collins RA, Howard CJ, Duggan SE, Werling D (1999). Bovine interleukin-12 and modulation of IFNγ production. Vet Immunol Immunopathol.

[38] Wattegedera S, Sills K, Howard CJ, Hope JC, McInnes CJ, Entrican G (2004). Variability in cytokine production and cell proliferation by mitogen-activated ovine peripheral blood mononuclear cells: modulation by interleukin (IL)-10 and IL-12. Vet Immunol Immunopathol.

[39] Rozen S, Skaletsky H (1999). Primer3 on the WWW for general users and for biologist programmers. Methods Mol Biol.

[40] Pfaffl MW (2001). A new mathematical model for relative quantification in real-time RT–PCR. Nucleic Acids Res.

[41] Jensen K, Talbot R, Paxton E, Waddington D, Glass E (2006). Development and validation of a bovine macrophage specific cDNA microarray. BMC Genomics.

[42] Ziegler-Heitbrock H (2000). Definition of human blood monocytes. J Leukoc Biol.

[43] Sallusto F, Cella M, Danieli C, Lanzavecchia A (1995). Dendritic cells use macropinocytosis and the mannose receptor to concentrate macromolecules in the major histocompatibility complex class II compartment: downregulation by cytokines and bacterial products. J Exp Med.

[44] Werling D, Hope JC, Chaplin P, Collins RA, Taylor G, Howard CJ (1999). Involvement of caveolae in the uptake of respiratory syncytial virus antigen by dendritic cells. J Leukoc Biol.

[45] Gupta VK, McConnell I, Dalziel RG, Hopkins J (1996). Identification of the sheep homologue of the monocyte cell surface molecule - CD14. Vet Immunol Immunopathol.

[46] Boysen P, Storset AK (2009). Bovine natural killer cells. Vet Immunol Immunopathol.

[47] Ibeagha-Awemu E, Lee J-W, Ibeagha A, Zhao X (2008). Bovine CD14 gene characterization and relationship between polymorphisms and surface expression on monocytes and polymorphonuclear neutrophils. BMC Genet.

[48] Hedges SB, Dudley J, Kumar S (2006). TimeTree: a public knowledge-base of divergence times among organisms. Bioinformatics.

[49] Garcia-Morales C, Rothwell L, Moffat L, Garceau V, Balic A, Sang HM, Kaiser P, Hume DA (2014). Production and characterisation of a monoclonal antibody that recognises the chicken CSF1 receptor and confirms that expression is restricted to macrophage-lineage cells. Dev Comp Immunol.

[50] Moffat L, Rothwell L, Garcia-Morales C, Sauter KA, Kapetanovic R, Gow DJ, Hume DA (2014). Development and characterisation of monoclonal antibodies reactive with porcine CSF1R (CD115). Dev Comp Immunol.

[51] Ingersoll M, Spanbroek R, Lottaz C, Gautier EL, Frankenberger M, Hoffmann R, Lang R, Haniffa M, Collin M, Tacke F, Habenicht AJR, Ziegler-Heitbrock L, Randolph GJ (2010). Comparison of gene expression profiles between human and mouse monocyte subsets. Blood.

[52] Ziegler-Heitbrock L (2014). Monocyte subsets in man and other species. Cell Immunol.

[53] Ancuta P, Liu K-Y, Misra V, Wacleche V, Gosselin A, Zhou X, Gabuzda D (2009). Transcriptional profiling reveals developmental relationship and distinct biological functions of CD16+ and CD16- monocyte subsets. BMC Genomics.

[54] Sunderkotter C, Nikolic T, Dillon M, Van Rooijen N, Stehling M, Drevets D, Leenen P (2004). Subpopulations of mouse blood monocytes differ in maturation stage and inflammatory response. J Immunol.

[55] Nguyen TKA, Reinink P, Messlaki CE, Im JS, Ercan A, Porcelli SA, Rhijn IV (2015). Expression patterns of bovine CD1 in vivo and assessment of the specificities of the anti-bovine CD1 antibodies. PLoS One.

[56] Van Rhijn I, Nguyen TKA, Michel A, Cooper D, Govaerts M, Cheng T-Y, van Eden W, Moody DB, Coetzer JAW, Rutten V, Koets AP (2009). Low cross-reactivity of T-cell responses against lipids from Mycobacterium bovis and M. avium paratuberculosis during natural infection. Eur J Immunol.

[57] Howard CJ, Sopp P, Brownlie J, Kwong LS, Parsons KR, Taylor G (1997). Identification of two distinct populations of dendritic cells in afferent lymph that vary in their ability to stimulate T cells. J Immunol.

[58] Banchereau J, Steinman RM (1998). Dendritic cells and the control of immunity. Nature.

[59] Hope JC, Guzman E, Cubillos-Zapata C, Stephens SA, Gilbert SC, Prentice H, Sopp P, Howard CJ, Charleston B (2012). Migratory sub-populations of afferent lymphatic dendritic cells differ in their interactions with Mycobacterium bovis Bacille Calmette Guerin. Vaccine.

[60] Wollenberg A, Mommaas M, Oppel T, Schottdorf E-M, Gunther S, Moderer M (2002). Expression and function of the mannose receptor cd206 on epidermal dendritic cells in inflammatory skin diseases. J Invest Dermatol.

[61] Burgdorf S, Lukacs-Kornek V, Kurts C (2006). The mannose receptor mediates uptake of soluble but not of cell-associated antigen for cross-presentation. J Immunol.

[62] Chamorro S, Revilla C, Álvarez B, Alonso F, Ezquerra Á, Domínguez J (2005). Phenotypic and functional heterogeneity of porcine blood monocytes and its relation with maturation. Immunology.

[63] Chamorro S, Revilla C, Álvarez B, López-Fuertes L, Ezquerra Á, Domínguez J (2000). Phenotypic characterization of monocyte subpopulations in the pig. Immunobiology.

[64] Buechler C, Ritter M, Orsó E, Langmann T, Klucken J, Schmitz G (2000). Regulation of scavenger receptor CD163 expression in human monocytes and macrophages by pro- and antiinflammatory stimuli. J Leukoc Biol.

[65] Sulahian TH, Högger P, Wahner AE, Wardwell K, Goulding NJ, Sorg C, Droste A, Stehling M, Wallace PK, Morganelli PM, Guyre PM (2000). Human monocytes express CD163, which is upregulated by IL-10 and identical to p155. Cytokine.

[66] Noronha LE, Harman RM, Wagner B, Antczak DF (2012). Generation and characterization of monoclonal antibodies to equine CD16. Vet Immunol Immunopathol.

[67] Sánchez C, Doménech N, Vázquez J, Alonso F, Ezquerra A, Domínguez J (1999). The porcine 2a10 antigen is homologous to human cd163 and related to macrophage differentiation. J Immunol.

[68] Schäkel K, von Kietzell M, Hänsel A, Ebling A, Schulze L, Haase M, Semmler C, Sarfati M, Barclay AN, Randolph GJ, Meurer M, Rieber EP (2006). Human 6-sulfo lacnac-expressing dendritic cells are principal producers of early interleukin-12 and are controlled by erythrocytes. Immunity.

